# The antimicrobial peptide LL-37 triggers release of apoptosis-inducing factor and shows direct effects on mitochondria

**DOI:** 10.1016/j.bbrep.2021.101192

**Published:** 2021-12-20

**Authors:** Elisabeth Bankell, Xiaoyan Liu, Martin Lundqvist, Daniel Svensson, Karl Swärd, Emma Sparr, Bengt-Olof Nilsson

**Affiliations:** a Department of Experimental Medical Science, Lund University, BMC D12, SE-22184, Lund, Sweden; b Division of Physical Chemistry, Department of Chemistry, Lund University, SE-22100, Lund, Sweden; c Division of Biochemistry and Structural Biology, Department of Chemistry, Lund University, SE-22100, Lund, Sweden

**Keywords:** Apoptosis, Cathelicidin, Innate immunity, Mitochondria, Mitochondria model membranes

## Abstract

The human antimicrobial peptide LL-37 permeabilizes the plasma membrane of host cells, but LL-37-induced direct effects on mitochondrial membrane permeability and function has not been reported. Here, we demonstrate that LL-37 is rapidly (within 20 min) internalized by human osteoblast-like MG63 cells, and that the peptide co-localizes with MitoTracker arguing for accumulation in mitochondria. Subcellular fractionation and Western blot disclose that stimulation with LL-37 (8 μM) for 2 h triggers release of the mitochondrial protein apoptosis-inducing factor (AIF) to the cytosol, whereas LL-37 causes no release of cytochrome C oxidase subunit IV of the inner mitochondrial membrane, suggesting that LL-37 affects mitochondrial membrane permeability in a specific manner. Next, we investigated release of AIF and cytochrome C from isolated mitochondria by measuring immunoreactivity by dot blot. The media of mitochondria treated with LL-37 (8 μM) for 2 h contained 50% more AIF and three times more cytochrome C than that of control mitochondria, showing that LL-37 promotes release of both AIF and cytochrome C. Moreover, in vesicles reflecting mitochondrial membrane lipid composition, LL-37 stimulates membrane permeabilization and release of tracer molecules. We conclude that LL-37 is rapidly internalized by MG63 cells and accumulates in mitochondria, and that the peptide triggers release of pro-apoptotic AIF and directly affects mitochondrial membrane structural properties.

## Highlights

• LL-37 is internalized by osteoblast-like MG63 cells
• LL-37 accumulates in mitochondria
• LL-37 triggers release of apoptosis-inducing factor from mitochondria
• LL-37 permeabilizes mitochondrial membranes

## 1 Introduction

The human antimicrobial peptide LL-37 is produced by white blood cells and epithelial cells, and it shows activity against both gram-positive and gram-negative bacteria [[1], [2], [3]]. White blood cells and epithelial cells synthesize and export hCAP18 which is the pro-form of LL-37, and this protein is processed extracellularly by serine protease 3 and kallikrein 5 to biologically active LL-37 [4,5]. LL-37 shows antibacterial activity through permeabilization of the bacterial cell wall and via binding and neutralization of bacterial endotoxins [2,6,7]. Importantly, LL-37 permeabilizes not only bacterial cell walls but also the plasma membrane of human host cells [8].

High concentrations (>4 μM) of LL-37 have been shown to be cytotoxic to many different human cell types, and this effect correlates negatively with cellular levels of the globular C1q receptor (p33) protein [8]. The p33 protein shows high affinity for LL-37, and, interestingly, p33 is detected in mitochondria, suggesting that this pool of the protein may antagonize effects of LL-37 on mitochondrial structure and function [8]. It is well-documented that LL-37-induced host cell cytotoxicity involves caspase-independent apoptosis as demonstrated in human lung carcinoma cells, Jurkat T-cells and osteoblast-like MG63 cells [9,10]. In human cancer cells, Mader et al. [11] demonstrated that LL-37-induced caspase-independent apoptosis involves release of the mitochondrial protein apoptosis-inducing factor (AIF) from mitochondria to cytosol, and translocation of AIF from cytosol to nucleus. Notably, AIF is well-recognized as a mediator of caspase-independent apoptosis [12]. Although, Mader et al. suggest that LL-37 releases AIF from mitochondria via an indirect mechanism, they do not rule out the possibility that LL-37 also may have direct effects on mitochondria [11]. Hence, there are many signs indicating that LL-37-induced host cell cytotoxicity involves LL-37-mediated effects on mitochondrial structure and function.

In the present study, we assess effects of LL-37 on mitochondria in MG63 cells. We have previously demonstrated that LL-37 reduces viability and induces caspase-independent apoptosis in MG63 cells, effects which are associated with internalization of LL-37 by these cells [10,13]. High concentrations of LL-37 (∼ 1 μM), relevant for LL-37-induced attenuation of osteoblast viability, have been demonstrated in the gingival crevicular fluid collected at diseased sites in patients suffering from periodontitis [14,15]. In periodontitis the alveolar bone is degraded, and the teeth thereby lose their attachment. In fact, the concentration of LL-37 may be even higher locally in the tissue facing the osteoblasts than in the gingival crevicular fluid of periodontal pockets. Hence, it is of *in vivo* relevance to study the impact of LL-37 on MG63 cell structure and function.

The aim of this study was to investigate the hypothesis that LL-37 directly interacts with mitochondria and triggers release of AIF. We demonstrate that LL-37 is rapidly internalized by MG63 cells, and that the peptide accumulates in mitochondria. Furthermore, we show for the first time that LL-37 stimulates release of AIF from isolated mitochondria and releases tracer molecule from vesicles with similar lipid composition as endogenous mitochondria, suggesting that LL-37 directly affects mitochondrial membrane structure.

## 2 Material and methods

### 2.1 Cells and cell culture

The human osteoblast-like MG63 cell line was purchased from ATCC. Cells were cultured in DMEM/Ham's F12 (1:1, Life Technologies) supplemented with 10% fetal bovine serum (Biochrom GmbH) and antibiotics (penicillin 50 U/ml and streptomycin 50 μg/ml, both from Biochrom GmbH) and kept in a water-jacked cell incubator (37 °C, 5% CO_2_ in air). The cells were trypsinized after reaching confluence, counted using a LUNA automated cell counter (Logos Biosystem) and re-seeded twice a week.

### 2.2 Immunocytochemistry

Cells were cultured on coverslips, treated with or without LL-37 (4 μM) for 20 or 60 min at 37 °C and then stained with the fluorescent mitochondria-specific dye MitoTracker (MitoTracker Orange CM-H_2_TMRos, Molecular Probes, 500 nM) for 45 min in accordance with the manufacturer's instructions. LL-37 had no effect on cell morphology, indicating that it does not impair cell viability under these experimental conditions. The cells were washed with phosphate-buffered saline (PBS, Gibco), fixed in 4% paraformaldehyde and permeabilized using 0.2% Triton X-100. Unspecific binding sites were blocked with 2% bovine serum albumin. Cells were incubated with a mouse monoclonal LL-37 antibody (1:400) for 2 h [16], washed with PBS and incubated with a secondary goat anti-mouse Alexa Fluor 488 antibody (Thermo Fisher Scientific) at a dilution of 1:500. The LL-37 antibody is well characterized and previously used for both immunocytochemistry and Western blot, and it recognizes endogenous hCAP18 and LL-37 at their respective correct molecular weights and visualizes cellular expression of hCAP18/LL-37 [16,17]. Cells were washed and coverslips mounted on glass slides using Fluoroshield DAPI mounting medium (Sigma Aldrich). DAPI was included as a nuclear marker. DAPI and MitoTracker fluorescence and immunoreactivity for LL-37 were analyzed using a fluorescence microscope (Olympus BX60, Olympus) equipped with a digital camera (Olympus DP72, Olympus).

### 2.3 Subcellular fractionation and Western blot

Subcellular fractionation was performed according to Dimauro et al. [18]. For each sample, cells (∼20 millions) were scraped-off and homogenized 80 times on ice using a Dounce homogenizer. Homogenates were vortexed and centrifuged according to the protocol and nuclei, cytosol and mitochondria isolated. Cytosolic proteins were precipitated in acetone. Nuclear and mitochondrial proteins were extracted using sonication. The subcellular fractions were mixed with SDS-buffer (1:1) and boiled prior to Western blot analysis. Total protein concentration was determined using Bio-Rad DC Protein Assay (Bio-Rad) to ensure equal loading. For each lane 30 μg protein was loaded. Proteins were separated by SDS-PAGE on Criterion TGX 4–15% precast gels (Bio-Rad) and transferred to nitrocellulose membranes using Trans-Blot Turbo Transfer System (Bio-Rad). The membranes were blocked for 2 h with 1% casein in TBS (1:1) and incubated with primary histone H3 (Cell Signaling, #9715, rabbit, 1:2000, nuclear marker), glyceraldehyde 3-phosphate dehydrogenase (GAPDH) (Merck Millipore, #MAB374, mouse, 1:3000, cytosolic marker) and cytochrome C oxidase subunit IV (COX IV) (Cell Signaling, #4844, rabbit, 1:1000, mitochondrial marker) antibodies at 4 °C overnight and for 3 days at 4 °C with AIF primary antibody (Santa Cruz Biotechnology, #sc-13116, mouse, 1:1000, mitochondrial marker). The immunoreactive bands were detected using horseradish peroxidase-conjugated secondary anti-mouse and anti-rabbit IgG (Cell Signaling, #7076S and #7074S, 1:5000) and SuperSignal West Femto chemiluminescence reagent (Thermo Fisher Scientific). The immunoreactive signals were acquired and analyzed using a LI-COR Odyssey Fc instrument (LI-COR Biosciences).

### 2.4 Dot blot

Mitochondria were isolated as described above and incubated in PBS at 37 °C with or without LL-37. At the end of incubation, mitochondria and conditioned medium were separated by centrifugation (11000 ***g***, 10 min, 4 °C) and supernatants collected. For dot blot, 1 μl medium was loaded onto nitrocellulose membranes. Membranes were blocked in TBS and 1% casein (1:1) for 2 h, and then incubated for 3 days at 4 °C with AIF or cytochrome C antibodies (Santa Cruz Biotechnology, #sc-13116, mouse, diluted 1:1000 and BD Biosciences, #556432, mouse, diluted 1:100). AIF and cytochrome C immunoreactivities were detected using a horseradish peroxidase-conjugated secondary anti-mouse IgG (Cell Signaling, #7076S, 1:5000) and SuperSignal West Femto chemiluminescence reagent (Thermo Fisher Scientific). Immunoreactive signal was acquired and analyzed by densitometric scanning using the LI-COR Odyssey Fc instrument.

### 2.5 Measurement of membrane leakage in giant lipid vesicles

Giant unilamellar vesicles (GUVs) were prepared on indium tin oxide (ITO)-coated coverslips (30–60 Ohms/Sq, Sigma-Aldrich) by electroformation in a microfluidic channel [19,20]. Stock solutions of lipids, POPC, POPC/CL (molar ratio 90/10), POPC/POPE/CL (molar ratio 60/30/10), were prepared in chloroform/methanol (volume ratio 9:1) at 0.2 mg/ml and supplemented with the fluorescent lipid analogue Liss Rhod PE (0.5 mol%, red). The ITO-coated coverslips were cleaned with ethanol, dried by nitrogen gas, and then lipid solution was deposited onto the conductive side of coverslips. They were dried in a vacuum chamber overnight, and thereafter mounted to a self-adhesive underside of microchannels (Ibidi Sticky-Slide VI 0.4). Another ITO-coated coverslip was attached to the top side of the microchannel, and both electrode coverslips were then connected to the frequency generator. PBS was added to the microfluidic channel and an AC-electric field (10 Hz, 3 V) applied for 3 h to generate GUVs. Fluorescence was assessed by an inverted confocal laser scanning microscope (CLSM, Leica SP5) at room temperature. After inspecting the GUVs alone, Alexa488 (2 μM) was added to the microfluidic channel. LL-37 was added stepwise at final concentrations ranging from 1 to 10 μM. For proper mixing, the microfluidic channel cell was gently rotated several times.

### 2.6 Measurement of membrane leakage in small lipid vesicles

Membrane leakage in small lipid vesicles was assessed as described by Barbet et al. [21]. To prepare small lipid vesicles, aliquots of lipid stock solutions (POPC/CL, molar ratio 90/10) were added to glass vials, solvent removed by evaporation, and then glass vials were placed under vacuum overnight. The buffer was prepared by dissolving carboxyfluorescein (CF) in PBS buffer to a final concentration of 65 mM CF in 120 mM PBS (0.8xPBS, pH 7.2) and pH adjusted to 7.2. Lipid films were dispersed in the PBS-CF buffer to a lipid concentration of 1 mM and extruded to unilamellar vesicles through a 0.1 μm syringe filter (Polycarbonate membranes). To remove excess CF in bulk solution, samples were added on an illustra^TM^ NAP^TM^-5 Column Sephadex^TM^ G-25 DNA Grade equilibrated with 150 mM PBS, which matches the total solute concentration (all type of ions and CF) inside the vesicle to avoid osmotic imbalance. The eluate was added to a new column of the same type, and the whole procedure was repeated twice. Samples were diluted to 10 μM in PBS, and transferred to a costar 96 well assay plate, (Corning). Fluorescence was assessed in a BMG FLUOstar plate reader, equipped with an injection syringe, excitation filter 485 nm and emission filter 520 nm, at 28 °C. Fluorescence was monitored every 0.2 s during 40 s. LL-37 of various concentrations was injected into the vesicle solution, giving final LL-37 concentration between 0.02 and 6.7 μM. Maximal fluorescence intensity was obtained by adding Triton X-100 (final concentration 1%) to samples. Control experiments were conducted by injecting PBS buffer. CF concentration in the sample was adjusted so that it falls within concentration regime where the fluorescence intensity is proportional to the concentration of released dye. All data were corrected by subtracting background fluorescence. To analyze vesicle size before and after leakage experiments, samples were further analyzed with Nanoparticle Tracking Analysis (NTA) using a NanoSight LM-10 instrument.

### 2.7 Agents

Synthetic LL-37 was purchased from Bachem AG and dissolved in DMSO. DMSO was included as vehicle control as appropriate. 1-palmitoyl-2-oleoyl-glycero-3-phosphocholine (POPC, C_42_H_82_NO_8_P), 1-palmitoyl-2-oleoyl-sn-glycero-3-phosphoethanolamine (POPE, C_39_H_76_NO_8_P), 1′,3′-bis[1,2-dioleoyl-sn-glycero-3-phospho]-glycerol (sodium salt), (Cardiolipin, CL). 1,2-dioleoyl-sn-glycero-3-phosphoethanolamine-N-(lissamine rhodamine B sulfonyl) (ammonium salt) (Liss Rhod PE, _C68H109N4O14PS2_) were purchased from Avanti Polar Lipids. Alexa Fluor™ 488 NHS Ester (C_25_H_15_Li_2_N_3_O_13_S_2_, Alexa488) was purchased from Thermo Fisher Scientific, and 5(6)-Carboxyfluorescein, ≥95% (HPLC), was purchased from Sigma-Aldrich. Milli-Q water was used for all experiments.

### 2.8 Statistics

For each experiment, the number of independent experiments and technical replicates are specified in the figure legend. Summarized data are presented as means ± S.E.M. Statistical significance was calculated by Student's two-tailed *t*-test for unpaired comparisons. P values less than 0.05 were regarded to show statistical significance.

## 3 Results

### 3.1 LL-37 is rapidly internalized by MG63 cells and accumulates in mitochondria

**Fig. 1:**
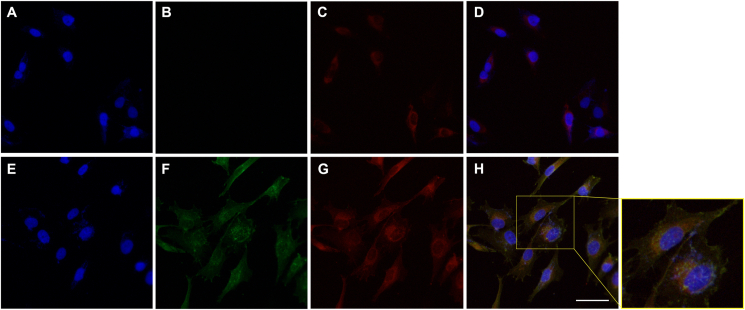
**LL-37 co-localizes with the mitochondrial marker MitoTracker in MG63 cells.** (**A-H**) Cells were treated with or without LL-37 (4 μM) for 60 min, and stained for nuclear marker DAPI (blue), LL-37 immunoreactivity (green) and MitoTracker (red). (**A-D**) Untreated control cells stained for (**A**) DAPI, (**B**) LL-37 immunoreactivity, (**C**) MitoTracker, and (**D**) overlay of LL-37 immunoreactivity and MitoTracker staining (yellow). (**E-H**) LL-37-treated cells stained with (**E**) DAPI, (**F**) for LL-37 immunoreactivity, (**G**) MitoTracker, and (**H**) overlay of LL-37 immunoreactivity and MitoTracker staining. For panel H, a magnification of two cells is shown. Pearson's correlation coefficient, r = 0.73 for LL-37 immunoreactivity and MitoTracker staining, was calculated in 60 cells using Image J. The bar in **H** represents 50 μm and applies to all images. Experiments were performed three times in duplicate. (For interpretation of the references to colour in this figure legend, the reader is referred to the Web version of this article.)

In the first experiments, we assessed uptake and distribution of exogenous LL-37 in MG63 cells by immunocytochemical analysis. Cells were treated with or without LL-37 (4 μM) for 60 min, and the same cells were triple stained for LL-37 (immuno), mitochondria (MitoTracker) and nuclei (DAPI) (Fig. 1A–H). In cells treated with synthetic LL-37, we observed cytoplasmic immunoreactivity for LL-37, showing that cells rapidly internalize the peptide (Fig. 1F). A very strong immunoreactive signal for LL-37 was observed in the perinuclear region of the cytoplasm, whereas nuclei were negative (Fig. 1F). No immunoreactivity for LL-37 was detected in untreated control cells, suggesting that MG63 cells express no endogenous LL-37 (Fig. 1B). MitoTracker staining was observed in cytoplasm of both untreated control cells and LL-37-treated cells (Fig. 1C and G). The MitoTracker staining was especially strong in the perinuclear region of cytoplasm (Fig. 1C and G). Co-localization of LL-37 immunoreactivity and MitoTracker staining was observed in the overlay (Fig. 1H). No immunoreactive signal was observed after omission of the primary LL-37 antibody (data not shown). Next, we assessed mitochondrial accumulation of exogenous LL-37 in MG63 cells treated with the peptide for a short time (20 min). Already after 20 min incubation with LL-37 (4 μM), we observed cytoplasmic immunoreactivity for LL-37 and the peptide co-localized with MitoTracker (Supplemental Fig. 1). Overall, the staining pattern and intensity for LL-37 immunoreactivity and MitoTracker was similar in cells treated with exogenous LL-37 for either 20 or 60 min indicating that the peptide rapidly accumulates in mitochondria.

### 3.2 Subcellular fractionation/Western blot discloses release of AIF from mitochondria to cytosol in MG63 cells

**Fig. 2:**
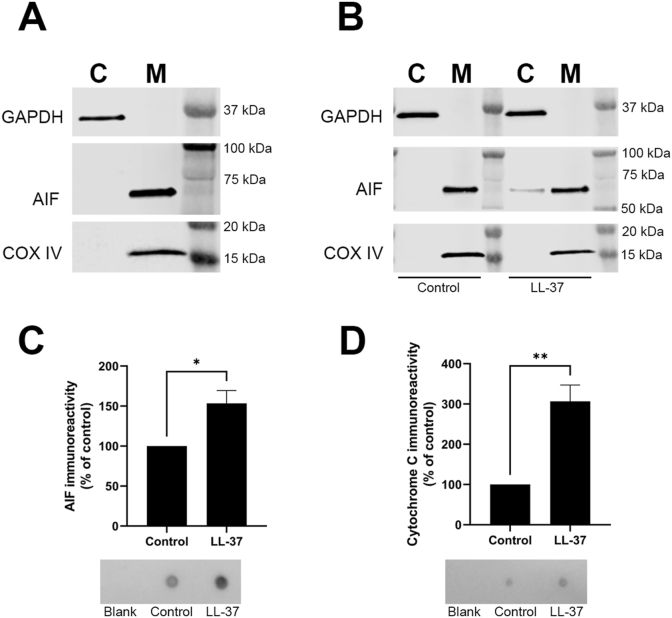
**LL-37 triggers release of mitochondrial AIF in MG63 cells.** (**A**) Western blot analysis shows immunoreactivity for GAPDH at its expected molecular weight of 37 kDa in cytosolic (C) but not mitochondrial (M) fractions, whereas AIF and COX IV immunoreactivity (expected molecular weight 57 and 17 kDa, respectively) is seen only in mitochondrial fractions demonstrating successful isolation and purification of cytosolic and mitochondrial proteins. (**B**) In cells stimulated with LL-37 (8 μM) for 2 h but not in control cells, an AIF immunoreactive band is clearly visible in cytosolic fractions. No cytosolic immunoreactivity for COX IV is observed either in LL-37-treated or control cells. Experiments were performed two times, and each sample was analyzed in triplicate. (**C, D**) Conditioned media were collected from mitochondria stimulated with or without LL-37 (8 μM) for 2 h. Immunoreactivity for AIF and cytochrome C in media was assessed by dot blot, showing that treatment with LL-37 increases AIF and cytochrome C by 50% and three times, respectively. Summarized data are presented as means ± S.E.M. of three independent experiments. For each experiment, control was set to 100%. Each sample was analyzed in triplicate. * and ** represent P < 0.05 and P < 0.01, respectively.

We hypothesize that LL-37 permeabilizes mitochondrial membranes thereby releasing mitochondrial proteins such as AIF to cytosol. Therefore, we performed subcellular fractionation/Western blot analysis to investigate if LL-37 triggers release of mitochondrial AIF and COX IV to cytosol. We used immunoreactivity against histone H3, GAPDH and AIF/COX IV as markers for nuclear, cytosolic, and mitochondrial fractions, respectively. As seen in Fig. 2A, we successfully isolated and purified cytosolic and mitochondrial fractions expressing respectively GAPDH or AIF and COX IV. Nuclear fractions contained histone H3 but also AIF and COX IV, indicating that some mitochondria/mitochondrial remnants were redistributed to nuclei during early steps of fractionation, e.g. during homogenization and/or centrifugation, and hence nuclear fractions were discarded (data not shown). In cells treated with LL-37 (8 μM) for 2 h, an immunoreactive band for AIF was detected in the cytosolic fraction, whereas no or very weak AIF immunoreactivity was observed in the cytosol of control cells (Fig. 2B). In order to obtain a high enough concentration of LL-37 to affect mitochondria without unwanted effects on cell viability, we used 8 μM LL-37 for these experiments. Immunoreactivity for COX IV was observed in mitochondrial but not in cytosolic fractions of LL-37-treated cells, showing that LL-37 causes a relatively mild permeabilization of mitochondria which does not affect inner membranes where COX IV is localized (Fig. 2B). Thus, these data show that LL-37 is rapidly internalized and that the peptide releases mitochondrial AIF.

### 3.3 Dot blot analysis shows that LL-37 triggers release of AIF and cytochrome C from MG63 cell mitochondria

Next, we isolated mitochondria, dispersed them in PBS, and treated the mitochondria with or without LL-37 (8 μM) for 2 h, and then collected their conditioned media. We analyzed AIF and cytochrome C contents in media with dot blot. Immunoreactivity for AIF was observed both in media of control mitochondria and in those of LL-37-stimulated mitochondria indicating a spontaneous release of AIF (Fig. 2C). However, importantly, the immunoreactive signal was about 50% higher in media of LL-37-treated mitochondria compared to that of control mitochondria, showing that LL-37 triggers release of AIF from isolated mitochondria (Fig. 2C). In media of LL-37-treated mitochondria, we detected about three times higher levels of cytochrome C compared to that of control mitochondria, showing that LL-37 also stimulates release of mitochondrial cytochrome C (Fig. 2D).

### 3.4 Membrane leakage induced by LL-37

**Fig. 3:**
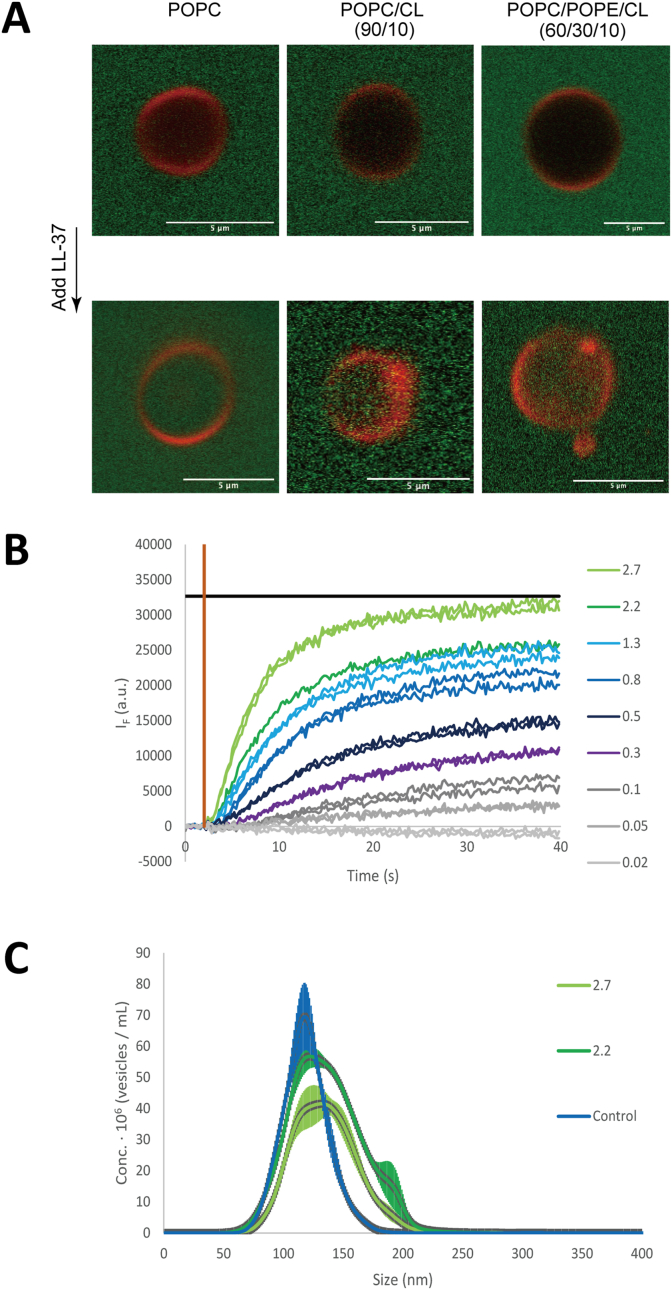
**LL-37 causes mitochondrial membrane leakage. (A)** Giant vesicles with different lipid composition assessed by laser scanning confocal microscopy before (upper panels) and after (lower panels) introduction of LL-37 (1, 2, 3, 5 and 8 μM). Images with overlapping signals show empty vesicles (red) with Alexa488 (green) on the outside (upper panels). Upon LL-37 treatment (>5 μM), Alexa488 enters the vesicles (lower panels). (**B**) Leakage of small lipid vesicles (POPC/CL, molar ratio 90/10) induced by LL-37 was monitored by CF fluorescence. Numbers in the figure refer to concentration (μM) of LL-37. The brown vertical line indicates timepoint at which the peptide was injected into the sample. The black horizontal line represents maximal leakage detected by Triton X-100. (**C**) Size distribution of vesicles was characterized using Nanoparticle Tracking Analysis. Numbers in the figure refer to concentration (μM) of LL-37. Experiments were performed three times. Two individual samples were analyzed for each concentration of LL-37. (For interpretation of the references to colour in this figure legend, the reader is referred to the Web version of this article.)

Finally, we investigated whether LL-37 may act on the lipid membrane itself, and thereby permeabilize membranes containing lipids relevant to mitochondria membranes [22]. For this purpose, we prepared model lipid vesicles that were either giant unilamellar vesicles (GUV's, ∼ 5 μm in diameter) or small lipid vesicles (∼ 100 nm in diameter) and studied their membrane integrity. Fig. 3A shows representative images of GUV's composed of either POPC, POPC/CL or POPC/POPE/CL together with a tracer amount of a red fluorescent lipid analogue Liss-Rh PE before and after addition of LL-37 (1, 2, 3, 5 and 8 μM). The integrity of GUVs is demonstrated by inability of Alexa488 (green) to penetrate the vesicles. Addition of LL-37 (>5 μM) to GUVs resulted in appearance of Alexa488 inside the vesicles, and similar effects were found for all model lipid systems investigated (Fig. 3A).

In order to get quantitative measurements of LL-37-induced membrane leakage, we performed bulk experiments to study release of an encapsulated dye, CF, from small unilamellar vesicles (Fig. 3B). Inside vesicles, CF is present in very high concentration, and this leads to self-quenching (no/low fluorescence). When the dye is released from the vesicles, the solution is diluted to concentrations low enough to avoid self-quenching and thereby to fluorescence signal. LL-37-induced leakage was followed over time showing that LL-37 triggered, in a concentration-dependent manner, a rapid release of CF (Fig. 3B). The size of vesicles was analyzed before and after the leakage experiments (Fig. 3C). For all conditions shown in Fig. 3, the majority of vesicles remains in solution also after addition of LL-37, implying that LL-37 indeed induces membrane permeabilization. At high concentrations of the peptide (>2.7 μM), LL-37 seems to cause complete rupture of vesicles, which also contributes to release of dye (Supplemental Fig. 2).

## 4 Discussion

Here, we provide evidence, by using both whole cells and isolated mitochondria, that the antimicrobial peptide LL-37 mediates release of pro-apoptotic AIF from mitochondria of human osteoblast-like MG63 cells, and moreover, we show that LL-37 affects mitochondrial membrane structure through a direct mechanism at concentrations which are relevant for LL-37-induced cytotoxicity *in vivo* [8,15]. We show that the peptide is rapidly internalized and accumulates in mitochondria, where it triggers release of AIF to the cytosol. In human macrophages, internalization of LL-37 by endocytosis seems to be partially dependent on the P2X_7_ receptor, but other mechanisms are probably also involved in cellular import of the peptide [23]. Importantly, we also demonstrate that LL-37 enhances release of AIF from isolated MG63 cell mitochondria. Additionally, we show that LL-37 enhances release of tracer molecules from vesicles with a composition of lipids similar to endogenous mitochondria, suggesting that LL-37 directly interacts with mitochondrial membranes and affect their properties. Interestingly, the vesicles used here by us contain lipids but not proteins, providing evidence that LL-37 interacts with lipids. Thus, we propose that exogenous LL-37 accumulates in mitochondria and permeabilizes mitochondrial membranes via a direct mechanism.

Previously, it has been demonstrated that LL-37 increases release of cytosolic LDH by human host cells, suggesting that LL-37 permeabilizes the plasma membrane [8,13]. It is well-known that LL-37 disrupts bacterial model lipid bilayers and shows anti-bacterial activity through permeabilization of bacterial cell walls [1,7,24]. Hence, LL-37 increases permeability of both human host cell plasma membranes and bacterial cell walls, but it also influences permeability of organelles such as mitochondria. We suggest, based on the present findings, that LL-37 permeabilizes mitochondria and promotes release of AIF which may drive apoptosis. Interestingly, release of mitochondrial AIF to the cytosol and translocation of this factor to nuclei triggers DNA fragmentation, and AIF has been shown to be critical for LL-37-induced caspase-independent human cancer cell apoptosis [11].

LL-37 releases AIF and cytochrome C but not COX IV, indicating that LL-37-induced effects on mitochondrial structure/function are relatively mild and probably only involve the outer mitochondrial membrane. Importantly, both AIF and cytochrome C are believed to be situated in the intermembrane space of the mitochondria, whereas COX IV is localized to the inner membrane [12]. COX IV is a well-known marker for mitochondrial integrity, and it has been reported to remain in the mitochondria of human leukemia cells treated with a high concentration (40 μM) of LL-37 [11]. Hence, it seems that LL-37 acts through a direct and specific mechanism involving permeabilization of the outer mitochondrial membrane and subsequent release of pro-apoptotic AIF, whereas it causes no complete disintegration of mitochondrial structure.

Mitochondria are supposed to have arisen from bacteria and considered to share structural and functional properties with bacteria [25]. It is well-recognized that the sensitivity of bacteria to LL-37 varies between different bacterial strains [26,27]. Variations in anti-bacterial activity of LL-37 probably reflect differences in LL-37-induced interactions with bacterial cell walls. Here, we show that LL-37 promotes release of mitochondrial AIF, raising the interesting hypothesis that mitochondrial membranes show structural and functional similarity with bacterial cell walls of bacteria sensitive to LL-37.

## Declaration of interest

The authors declare that there are no conflicts of interest.

## Funding

This study was supported by grants from the 10.13039/501100005390Alfred Österlund Foundation, the 10.13039/100016409Gyllenstiernska Krapperups Foundation, the Heart-Lung Foundation (20200222) and the 10.13039/501100004359Swedish Research Council (2020-00908).

## Declaration of competing interest

The authors declare that they have no known competing financial interests or personal relationships that could have appeared to influence the work reported in this paper.

## Data Availability

Data will be made available on request.

## Appendix A Supplementary data

Multimedia component 2 Multimedia component 2

figs1 figs1

figs2 figs2

figs3 figs3
