# Supplementary material for: The antimicrobial peptide LL-37 triggers release of apoptosis-inducing factor and shows direct effects on mitochondria
Source: Biochem Biophys Rep. 2021 Dec 20;29:101192. doi: 10.1016/j.bbrep.2021.101192 (PMC8695256; doi:10.1016/j.bbrep.2021.101192)
Supplement: Multimedia component 1 [file mmc1.docx]

**Supplemental Figure 1. Exogenous LL-37 accumulates in the mitochondria of MG63 cells already within 20 min.** (**A-H**) Cells were treated with or without LL-37 (4 µM) for 20 min, and the same cells were stained for DAPI (blue), LL-37 immunoreactivity (green) and MitoTracker (red). (**A-D**) Untreated control cells stained for (**A**) DAPI, (**B**) LL-37 immunoreactivity, (**C**) MitoTracker, and (**D**) overlay of LL-37 immunoreactivity and MitoTracker staining (yellow). (**E-H**) LL-37-treated cells stained for (**E**) DAPI, (**F**) LL-37 immunoreactivity, (**G**) MitoTracker, and (**H**) overlay of LL-37 immunoreactivity and MitoTracker staining. Pearson’s correlation coefficient, r=0.71 for LL-37 immunoreactivity and MitoTracker staining, was calculated in 56 cells using Image J. The bar in **H** represents 50 µm and applies to all images. Experiments were performed three times in duplicate.

**Supplemental Figure 2. LL-37 causes mitochondrial membrane leakage in a concentration-dependent manner and collapse of vesicles at high concentration.** (**A**) Leakage of small lipid vesicles (POPC/CL, molar ratio 90/10) induced by LL-37 was monitored by CF fluorescence. Numbers in the figure refer to concentration (µM) of LL-37. The brown vertical line indicates the timepoint at which the peptide was injected into the sample. The black horizontal line represents maximal leakage detected by Triton X-100. (**C**) Size distribution of vesicles was characterized using Nanoparticle Tracking Analysis. Numbers in the figure refer to concentration (µM) of LL-37. Experiments were performed three times. Two individual samples were analyzed for each concentration of LL-37, except for the highest one (6.7 µM) where four samples were included.
