# Supplementary material for: The antimicrobial peptide LL-37 triggers release of apoptosis-inducing factor and shows direct effects on mitochondria
Source: Biochem Biophys Rep. 2021 Dec 20;29:101192. doi: 10.1016/j.bbrep.2021.101192 (PMC8695256; doi:10.1016/j.bbrep.2021.101192)
Supplement: Multimedia component 2 [file mmc2.docx]

**Uncropped western blots-not for publication: Original uncropped western blots showing an immunoreactive band for AIF in the cytosolic fraction of cells treated with LL-37 (8 µM) for 2 h.** C and M stand for cytosolic and mitochondrial fraction, respectively. Representative blots are shown in figures 2A and 2B.
