# Supplementary figures and images for: The antimicrobial peptide LL-37 triggers release of apoptosis-inducing factor and shows direct effects on mitochondria
Source: Biochem Biophys Rep. 2021 Dec 20;29:101192. doi: 10.1016/j.bbrep.2021.101192 (PMC8695256; doi:10.1016/j.bbrep.2021.101192)

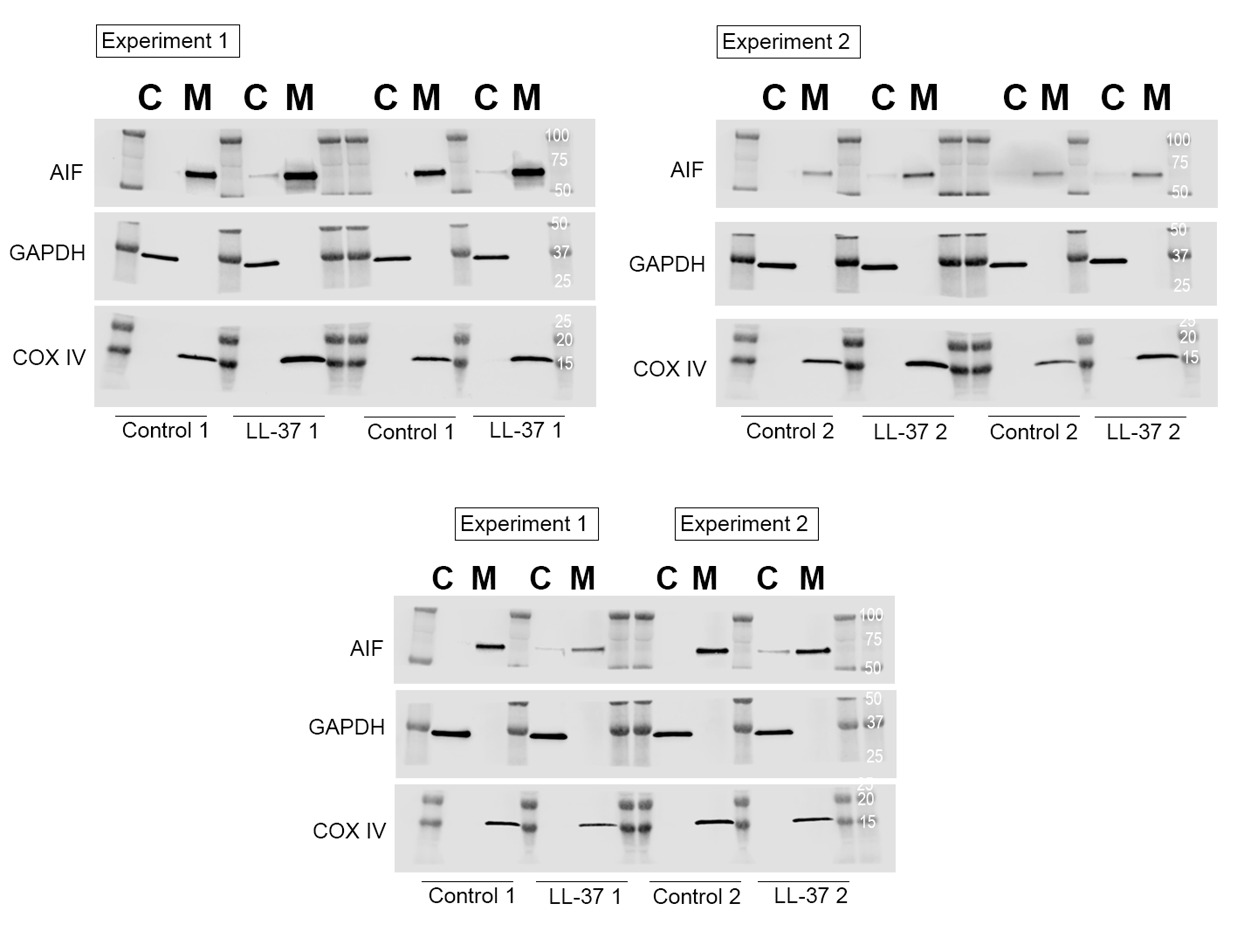

Supplement: figs1 [file mmcfigs1.jpg]

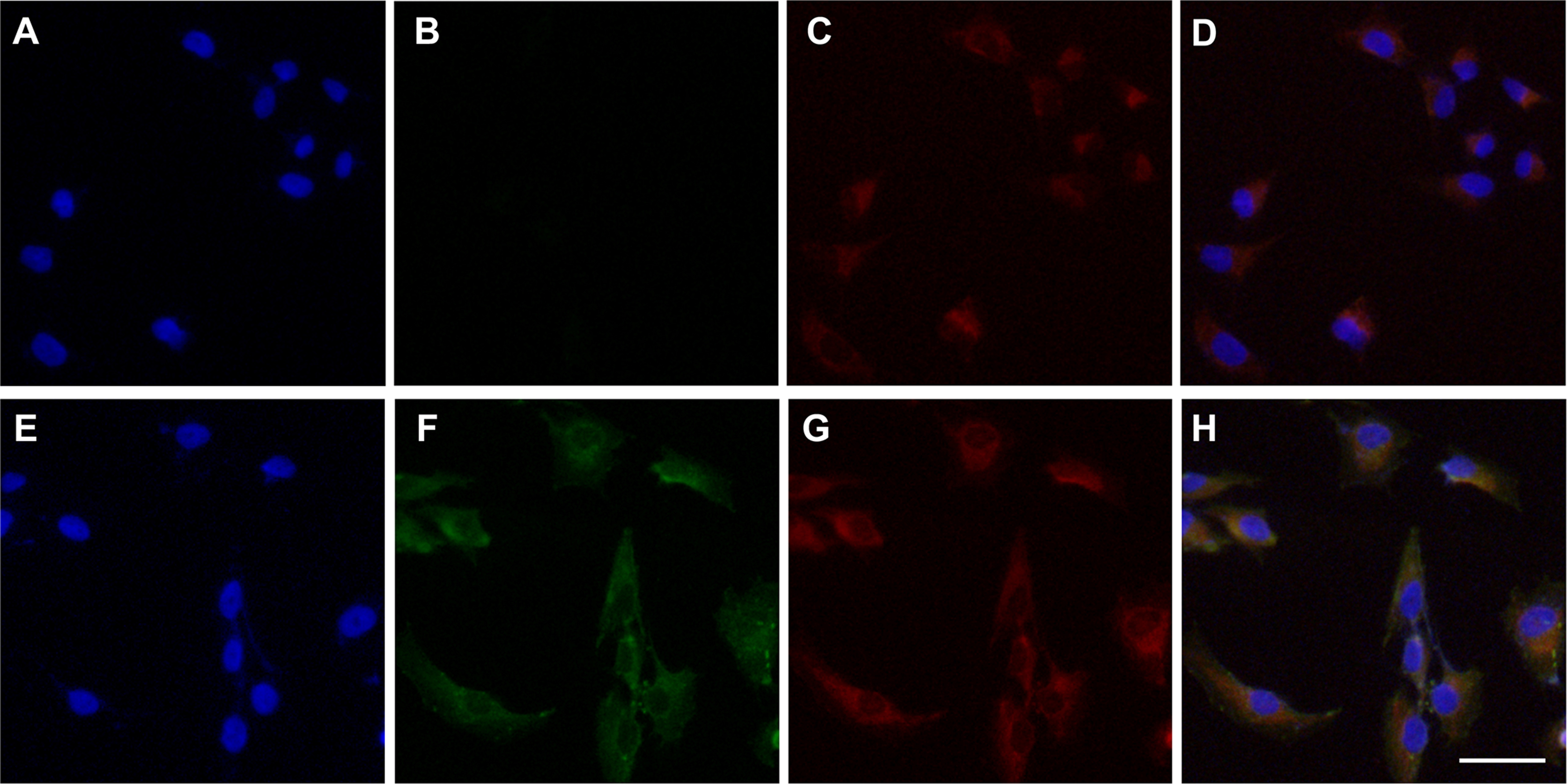

Supplement: figs2 [file mmcfigs2.jpg]

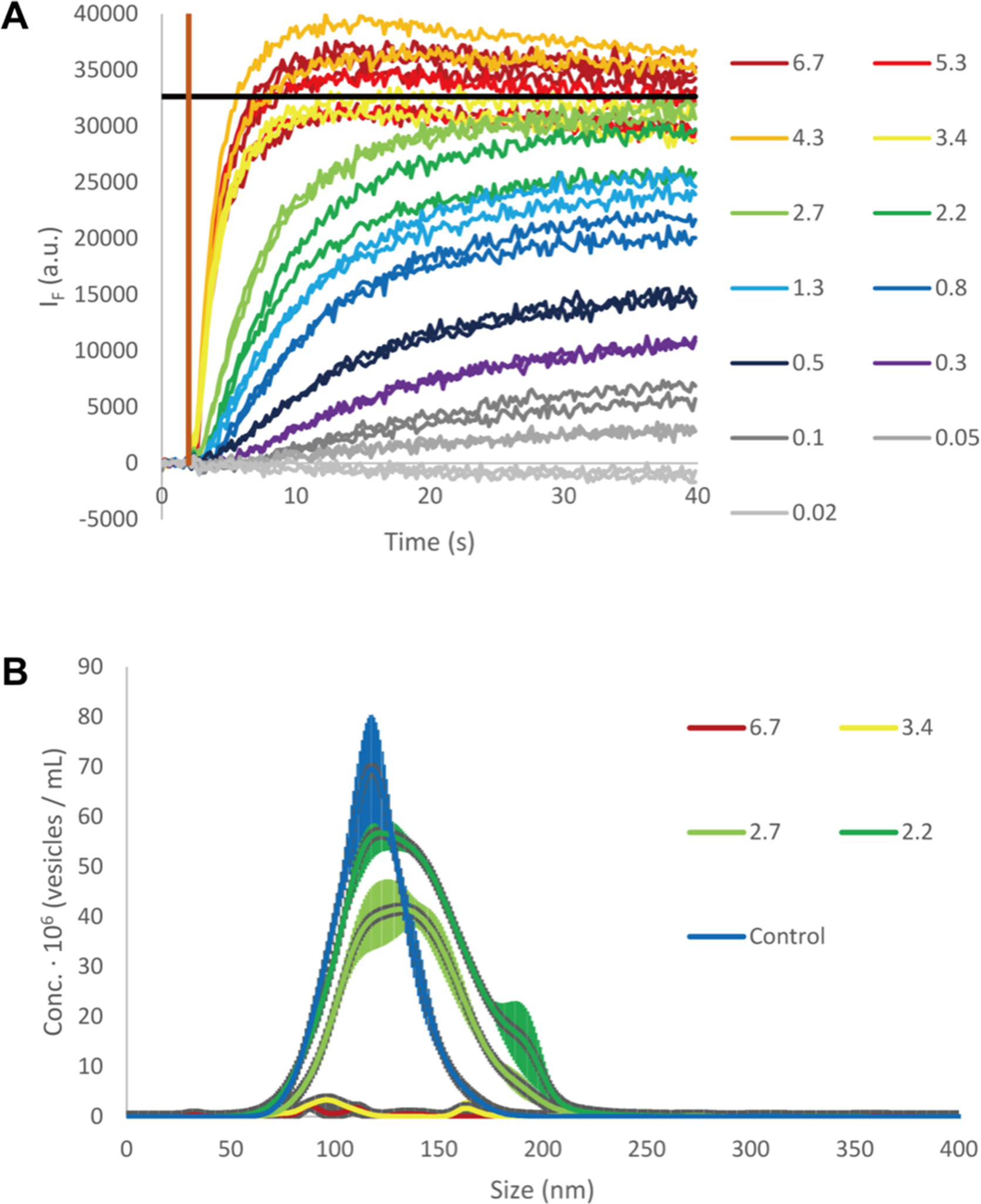

Supplement: figs3 [file mmcfigs3.jpg]
